# Genetic Interactions Between *Drosophila sialyltransferase* and *β1,4-N-acetylgalactosaminyltransferase-A* Genes Indicate Their Involvement in the Same Pathway

**DOI:** 10.1534/g3.112.001974

**Published:** 2012-06-01

**Authors:** Michiko Nakamura, Dheeraj Pandey, Vladislav M. Panin

**Affiliations:** Department of Biochemistry & Biophysics, Texas A&M University, College Station, Texas 77843-2128

## Abstract

Sialylated glycans play a prominent role in the *Drosophila* nervous system where they are involved in the regulation of neural transmission. However, the functional pathway of sialylation in invertebrates, including *Drosophila*, remains largely unknown. Here we used a combination of genetic and behavioral approaches to shed light on the *Drosophila* sialylation pathway. We examined genetic interactions between *Drosophila sialyltransferase* (*DSiaT*) and *β1,4-N-acetylgalactosaminyltransferase* (*β4GalNAcT*) genes. Our results indicated that *β4GalNAcTA* and *DSiaT* cooperate within the same functional pathway that regulates neural transmission. We found that *β4GalNAcTA* is epistatic to *DSiaT*. Our data suggest an intriguing possibility that β4GalNAcTA may participate in the biosynthesis of sialylated glycans.

Sialylation is a common type of protein glycosylation in vertebrate organisms (Schauer 2009; Varki and Schauer 2009). In mammals, sialylated glycans affect a plethora of protein interactions in the extracellular milieu, play a variety of important biological roles in development, and influence the physiology of many tissues and organs (Varki 2007, 2008). Sialylation is prominently enriched in the nervous system of vertebrates and is involved in crucial regulatory processes (Kleene and Schachner 2004; Varki 2008). At the same time, the role and biosynthesis of sialylated glycans in invertebrates is not well understood. Although glycoprotein sialylation is ubiquitous and abundant in mammalian organisms, it accounts for less than 0.1% of the total content of N-glycans in fruit flies (Aoki *et al.* 2007). Despite their exceedingly low amount, sialylated glycans have an important function in the *Drosophila* central nervous system (CNS). Recent studies of Drosophila sialyltransferase (DSiaT), the enzyme mediating the last step in the sialylation pathway, indicated that sialylation regulates neural transmission and development, while representing a tightly controlled process limited to a subset of CNS neurons (Koles *et al.* 2004; Repnikova *et al.* 2010). However, the low level of sialylation makes its biochemical investigation in *Drosophila* a challenging task (Aoki *et al.* 2007; Koles *et al.* 2007). Here we used a genetic strategy, combined with the knowledge of glycan structures identified on fly glycoproteins, to shed light on the sialylation pathway in *Drosophila*.

The structure of *Drosophila* N-linked glycans indicates that galactose residues (Gal) of LacNAc termini (Galβ1,4GlcNAc) serve as acceptors for sialylation (Aoki *et al.* 2007; Koles *et al.* 2007). Therefore, a galactosyltransferase attaching β1,4-linked Gal to N-glycans should be required for sialylation, and this enzyme is expected to cooperate with DSiaT in the regulation of neural transmission. However, so far no β1,4 galactosyltransferase (β4GalT) of this type has been identified in invertebrates. In mammalian cells, the corresponding Gal residues are added by one of the six β4GalTs (β4GalT1–6), the enzymes that function with apparent redundancy in modifying N-glycans (Hennet 2002). In *Drosophila*, the family of most closely related homologs of these β4GalTs consists of two enzymes, β1,4-N-acetylgalactosaminyltransferases A and B (β4GalNAcTA and β4GalNAcTB) (Haines and Irvine 2005). However, when assayed *in vitro*, these two glycosyltransferases exhibit substrate specificity different from that of vertebrate β4GalTs. Both of them prefer to transfer N-acetylgalactosamine (GalNAc) and synthesize LacdiNAc (GalNAcβ1,4GlcNAc) instead of LacNAc, whereas their ability to transfer Gal is low (Chen *et al.* 2007; Haines and Irvine 2005; Ramakrishnan and Qasba 2007). Despite the fact that β4GalNAcTA and β4GalNAcTB have similar *in vitro* activities, they have non-redundant functions *in vivo* (Chen *et al.* 2007; Haines and Irvine 2005; Haines and Stewart 2007; Stolz *et al.* 2008). The β4GalNAcTB enzyme modifies glycosphingolipids, and its function affects EGFR signaling during oogenesis (Chen *et al.* 2007; Stolz *et al.* 2008). Because β4GalNAcTA is capable of elongating βGlcNAc-termini of glycosphingolipids by adding β1,4-linked GalNAc *in vitro*, this glycosyltransferase may also have some role in glycosphingolipid biosynthesis *in vivo* (Chen *et al.* 2007; Johswich *et al.* 2009). However, this role is likely to be minor because *β4GalNAcTA* mutants have no discernable defects of glycosphingolipids, and the endogenous targets of β4GalNAcTA remain largely elusive (Chen *et al.* 2007; Johswich *et al.* 2009; Stolz *et al.* 2008). Mutations in β4GalNAcTA result in behavioral phenotypes, ultrastructural defects of muscles, and neuromuscular junction abnormalities (Chen *et al.* 2007; Haines and Irvine 2005; Haines and Stewart 2007).

Considering the close evolutionary relationship between β4GalNAcTA/B and vertebrate β4GalTs, we reasoned that these *Drosophila* enzymes might participate in the biosynthesis of N-linked glycans *in vivo*. This scenario entails a possibility that *β4GalNAcTA/B* is involved in the generation of glycan acceptors for DSiaT, and therefore, the mutations in these genes would affect DSiaT-mediated processes. Here we test this hypothesis using genetic and behavioral approaches.

## Materials and Methods

### *Drosophila* rearing and genetic stocks

Flies were reared in a temperature-controlled (25°) and humidity-controlled (37%) environment at diurnal light conditions. We used the following mutant alleles for *DSiaT* and *β4GalNAcTA/B* genes: *DSiaT^s23^*, *β4GalNAcTA^4.1^*, and *β4GalNAcTB^GT^*, designated here as *DSiaT*^–^, *β4GalNAcTA*^–^, and *β4GalNAcTB*^–^, respectively. These mutants represent loss-of-function alleles and were previously described (Haines and Irvine 2005; Repnikova *et al.* 2010). Double mutants *DSiaT^–^ β4GalNAcTA*^–^ were generated by recombination. The *DSiaT*^–^ and *β4GalNAcTA*^–^ single and double mutants were confirmed by genomic PCR and sequencing for the presence of corresponding mutations: the *DSiaT^s23^* allele includes two stop codons within the DSiaT coding region that truncate the encoded DSiaT protein sequence at positions Cys18 and Leu377 (Repnikova *et al.* 2010); the *β4GalNAcTA^4.1^* allele includes a 609 bp deletion that removes 113 bp upstream of the start codon along with the downstream region encoding the first 143 amino acids of β4GalNAcTA (Haines and Irvine 2005). The following PCR primers were used for genomic PCR reactions: for *DSiaT^s23^*, *St-gen-up (5′-TTAAGTGCGAGCTAAAGGTCAATGC-3′)* and *Sia-spe (5′-CAACTAGTAATCGCGCTCCTCTTCAGTAG-3′)*; for *β4GalNAcTA^4.1^*, *TA-P2* (*5′-TGCCGCTGCTGTCAGGAT-3′)* and *TA-P3* (*5′-AACGAAGCGATGAACTGTTTGAAT-3′)*. The *β4GalNAcTB^GT^* mutation was confirmed by genomic PCR reactions with two sets of primers that amplify the genomic region of *β4GalNAcTB* disrupted by gene targeting, as described in Haines and Irvine (2005). The presence of *β4GalNAcTB*^–^ was also corroborated by scoring the dorsal appendage fusion phenotype in homozygous mutants (Chen *et al.* 2007). The ectopic expression of *β4GalNAcTA* was induced using the UAS-GAL4 system (Brand *et al.* 1994) specifically in neurons (*β4GalNAcTA^Neuro^*) or in muscles (*β4GalNAcTA^Muscle^*) with *C155-Gal4* and *Dmef2-Gal4* drivers, respectively (Lin and Goodman 1994; Ranganayakulu *et al.* 1996). We used *w^1118^ Canton-S* as a wild-type control in our experiments.

### Heat-induced paralysis assays

We assayed five-day-old males for heat-induced paralysis using the previously described protocol (Repnikova *et al.* 2010). At least 20 flies were assayed for each data point. Unless indicated otherwise, the heat-shock assays were performed with individual flies at 38°.

### Statistical analysis

We used Student unpaired *t*-test with two-tailed distribution to assess the statistically significant differences between groups of related data.

## Results and Discussion

**Figure 1:**
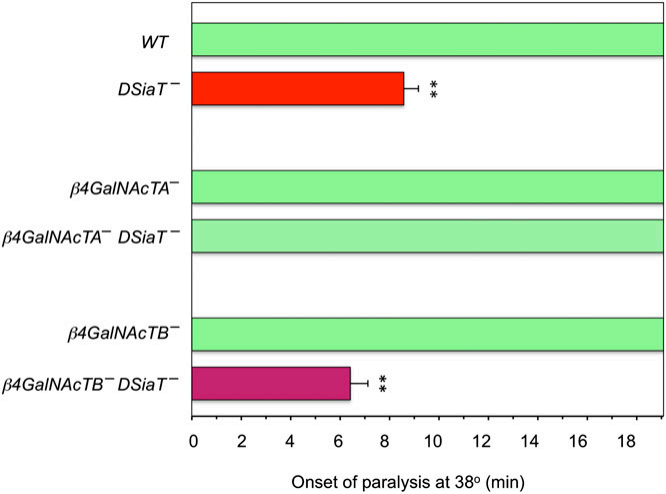
Heat-shock paralysis assay revealed epistatic interaction between *DSiaT* and *β4GalNAcTA*. At least 20 males were assayed for each genotype. *WT*, wild-type control (Canton S). Mutant alleles used in these experiments were *DSiaT^s23^*, *β4GalNAcTA^4.1^*, and *β4GalNAcTB^GT^* (Haines and Irvine 2005; Repnikova *et al.* 2010). ^**^Significant difference with *t*-test (*P* < 0.01). Error bars represent SEM.

**Figure 2:**
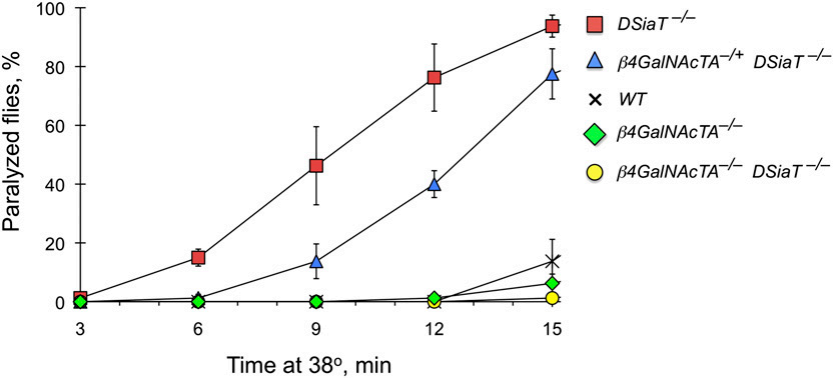
The kinetics of paralysis indicates that the *GalNAcTA* mutation semi-dominantly suppresses the phenotype of *DSiaT* mutants. The kinetics of paralysis was assayed using groups of 10 males as previously described (Repnikova *et al.* 2010). Each data point represents the average of three independent experiments. *WT*, wild-type control. Mutant alleles used in these experiments were *DSiaT ^s23^* and *β4GalNAcTA^4.1^*. Error bars represent SD.

**Figure 3:**
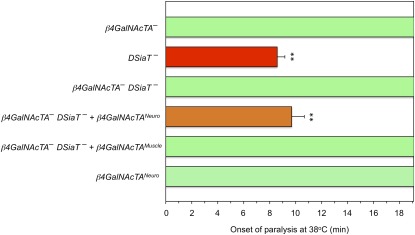
The neuronal function of *β4GalNAcTA* is required for the paralysis phenotype of *DSiaT* mutants. Transgenic expression of *β4GalNAcTA* in neurons of *β4GalNAcTA*^–^*DSiaT*^–^ double mutants could restore the paralysis phenotype, whereas the expression in muscles had no effect on the phenotype of the double mutants. Mutant alleles used in these experiments were *DSiaT^s23^* and *β4GalNAcTA^4.1^*. The ectopic expression of *β4GalNAcTA* was induced using UAS-GAL4 system specifically in neurons (*β4GalNAcTA^Neuro^*) or in muscles (*β4GalNAcTA^Muscle^*) with *C155-Gal4* and *Dmef2-Gal4* drivers, respectively. At least 20 males were assayed for each genotype. ^**^Significant difference with *t*-test (*P* < 0.01). Error bars represent SEM.

**Figure 4:**
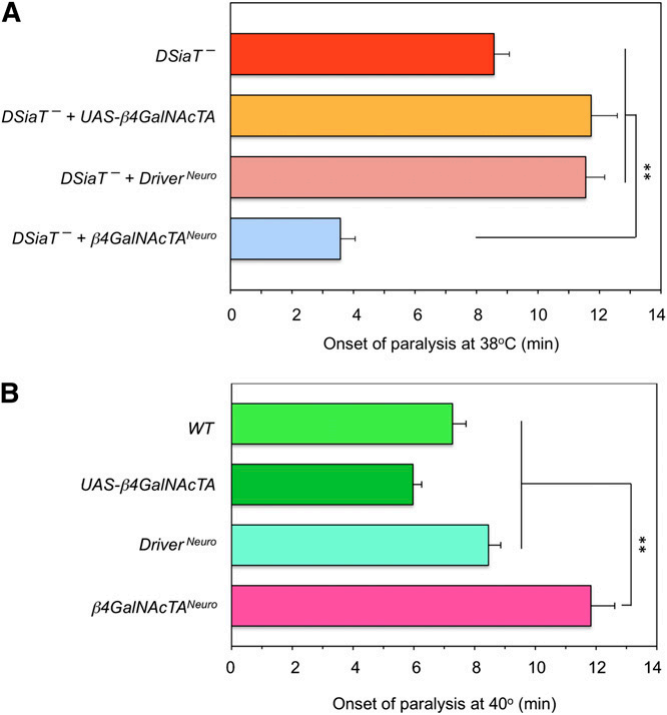
Ectopic expression of *β4GalNAcTA* in neurons exacerbates the heat-induced paralysis of *DSiaT* mutants (A) but alleviates it in wild-type flies (B). (A) The transgenic expression of *β4GalNAcTA* was induced in neurons of *DSiaT* mutants using *C155-GAL4* driver. *DSiaT* mutants with transgene alone (*DSiaT^–^ + UAS-β4GalNAcTA*) and driver alone (*DSiaT^–^ + Driver^Neuro^*) were also assayed as controls. (B) The transgenic expression of *β4GalNAcTA* was induced in neurons of wild-type flies using *C155-GAL4* driver. Genotypes with transgene alone (*UAS-β4GalNAcTA*) and driver alone (*Driver^Neuro^*) were also assayed as controls. To observe the heat-induced paralysis of wild-type flies, the heat-shock temperature was raised to 40°. Mutant alleles used in these experiments were *DSiaT^s23^* and ***β****4GalNAcTA^4.1^*. At least 20 males were assayed for each genotype. ^**^Significant difference with *t*-test (*P* < 0.01). Error bars represent SEM.

To test the possibility that β4GalNAcTA/B glycosyltransferases could be involved in the functional pathway mediated by sialylation, we examined genetic interactions between *DSiaT* and *β4GalNAcTA/B* genes. *DSiaT* mutations cause a characteristic temperature-sensitive paralysis phenotype (TS paralysis) (Repnikova *et al.* 2010). We used the TS paralysis assay to characterize genetic interactions between *DSiaT* and *β4GalNAcTA/B*. Whereas *DSiaT* mutants were consistently paralyzed within 7–10 min, neither *β4GalNAcTA* nor *β4GalNAcTB* mutants showed TS paralysis (Figure 1). Strikingly, the analysis of double mutants revealed that the *β4GalNAcTA* mutation suppressed the paralysis phenotype of *DSiaT* mutants. At the same time, no significant interaction was observed between *DSiaT* and *β4GalNAcTB* (Figure 1). To characterize the interaction between *DSiaT* and *β4GalNAcTA* in more detail, we assayed the “kinetics” of paralysis by counting the number of paralyzed flies in a 3-min interval after transferring them to the restrictive temperature (38°). We found that *β4GalNAcTA* mutation semi-dominantly suppressed the phenotype of *DSiaT* mutants, which indicates that the *DSiaT* phenotype is very sensitive to the level of *β4GalNAcTA* activity (Figure 2). It was previously shown that *β4GalNAcTA* plays separate roles in neural and muscle cells (Haines and Stewart 2007). Thus, we investigated whether the neural or muscle-specific function of *β4GalNAcTA* is responsible for the interaction with *DSiaT*. We used a rescue strategy with the UAS-GAL4 ectopic expression system (Brand *et al.* 1994) to induce the expression of β4GalNAcTA specifically in neurons or muscle cells of *DSiaT–β4GalNAcTA* double mutants. These experiments revealed that the neuronal expression of β4GalNAcTA could suppress the effect of *β4GalNAcTA* mutation on the paralysis of *DSiaT* mutants, whereas the expression in muscles did not influence the phenotype of the double mutants (Figure 3). Therefore, we concluded that it is the neuron-specific function of *β4GalNAcTA* that affects the paralysis of *DSiaT* mutants. Moreover, we found that the ectopic expression of β4GalNAcTA in the neurons of *DSiaT* mutants could further enhance the phenotype (Figure 4A), which again highlighted that the TS paralysis of *DSiaT* mutants depends on the neural activity of *β4GalNAcTA*. The involvement of neural activity of *β4GalNAcTA* in the *DSiaT*-mediated pathway is consistent with the fact that *DSiaT* function is restricted to neurons at all developmental stages (Koles *et al.* 2004; Repnikova *et al.* 2010). Collectively, our results indicate that *β4GalNAcTA* and *DSiaT* cooperate within the same functional pathway that regulates neural excitability and that *β4GalNAcTA* is epistatic to *DSiaT*.

The β4GalNAcTA protein is the closest *Drosophila* homolog of vertebrate β4GalT1–6 (Haines and Irvine 2005). These β4GalTs are thought to originate from invertebrate β4GalNAcTs during evolution (Haines and Irvine 2005; Ramakrishnan and Qasba 2007). Interestingly, the donor substrate specificity of β4GalT and β4GalNAcT enzymes, including β4GalNAcTA, can be changed between Gal and GalNAc just by a single amino acid substitution in the active site (Ramakrishnan and Qasba 2002, 2007). Moreover, the donor and acceptor specificities of mammalian β4GalT1 can be modified through the binding of a protein cofactor, α-lactalbumin (Do *et al.* 1995; Hennet 2002). Such “flexibility” of the β4GalT/β4GalNAcT catalytic pocket capable of adjusting to different substrates suggests that the β4GalNAcTA specificity may be modified *in vivo* by a co-factor to synthesize LacNAc termini of N-linked glycans. This possibility is further supported by the fact that the ability to bind a co-factor is an evolutionarily ancient feature of β4GalT/GalNAcT enzymes, and that this feature is also preserved for *Drosophila* β4GalNAcTA (Neeleman and van de Eijnden 1996; Ramakrishnan and Qasba 2007). The scenario that β4GalNAcTA may synthesize LacNAc termini, the potential acceptors for sialylation, is also consistent with the epistatic interaction between *β4GalNAcTA* and *DSiaT* that was revealed in our experiments. However, this scenario does not rule out that β4GalNAcTA has other functions that are not limited to its role in the DSiaT-mediated pathway. This is supported by the fact that β4GalNAcTA mutants exhibit some phenotypes apparently unrelated to the function of DSiaT, such as muscle abnormalities and the defects of neuromuscular junctions at muscle 6 during the larval stage (Haines and Stewart 2007; Repnikova *et al.* 2010).

The hypothesis that β4GalNAcTA may be involved in the biosynthesis of DSiaT acceptors predicts that the overexpression of β4GalNAcTA in the nervous system of wild-type flies, in the presence of endogenous DSiaT activity, may result in increased resistance to heat. This possibility is based on the fact that a limiting factor of the insect biosynthesis of complex N-glycans, including sialylated structures, is the high activity of the GlcNAcase *fused lobes* that could compete with galactosylation by removing GlcNAc from N-linked antennae prior to their elongation with Gal (Leonard *et al.* 2006; Watanabe *et al.* 2002). The upregulation of β4GalNAcTA might outcompete GlcNAcase, while protecting glycan termini from trimming by converting them to LacNAc, the substrate for further sialylation. Thus, in the presence of DSiaT, the overexpression of β4GalNAcTA may result in a more efficient biosynthesis of sialylated glycans, which in turn would increase the stability of neural transmission at elevated temperatures. Indeed, we observed that wild-type flies become more resistant to heat when β4GalNAcTA was ectopically overexpressed using a neuronal driver (Figure 4B).

Taken together our results demonstrated that *β4GalNAcTA* genetically interacts with *DSiaT*, indicating that these genes cooperate in the same functional pathway affecting neural transmission. Our data also suggest an intriguing possibility that β4GalNAcTA may participate *in vivo* in the biosynthesis of LacNAc termini of N-glycans, including sialylated glycans. In the light of the fact that the loss of *β4GalNAcTA* activity suppresses the mutant phenotype of DSiaT, it is tempting to speculate that sialic acids may play a masking role, capping LacNAc structures and thus regulating their interactions in the nervous system. However, other scenarios are also possible, and the mechanism of the interplay between β4GalNAcTA and sialylation pathway awaits further investigation using biochemical and *in vivo* approaches.

## References

[bib1] Aoki K. Perlman M. Lim J. M. Cantu R. Wells L., 2007 Dynamic developmental elaboration of N-linked glycan complexity in the Drosophila melanogaster embryo. J. Biol. Chem. 282: 9127–9142 17264077 10.1074/jbc.M606711200

[bib2] Brand A. H. Manoukian A. S. Perrimon N., 1994 Ectopic expression in Drosophila. Methods Cell Biol. 44: 635–654 7707973 10.1016/s0091-679x(08)60936-x

[bib3] Chen Y. W. Pedersen J. W. Wandall H. H. Levery S. B. Pizette S., 2007 Glycosphingolipids with extended sugar chain have specialized functions in development and behavior of Drosophila. Dev. Biol. 306: 736–749 17498683 10.1016/j.ydbio.2007.04.013

[bib4] Do K. Y. Do S. I. Cummings R. D., 1995 Alpha-lactalbumin induces bovine milk beta 1,4-galactosyltransferase to utilize UDP-GalNAc. J. Biol. Chem. 270: 18447–18451 7629170 10.1074/jbc.270.31.18447

[bib5] Haines N. Stewart B. A., 2007 Functional roles for beta1,4-N-acetlygalactosaminyltransferase-A in *Drosophila* larval neurons and muscles. Genetics 175: 671–679 17151241 10.1534/genetics.106.065565 PMC1800592

[bib6] Haines N. Irvine K. D., 2005 Functional analysis of Drosophila beta1,4-N-acetlygalactosaminyltransferases. Glycobiology 15: 335–346 15563714 10.1093/glycob/cwi017

[bib7] Hennet T., 2002 The galactosyltransferase family. Cell. Mol. Life Sci. 59: 1081–1095 12222957 10.1007/s00018-002-8489-4 PMC11337546

[bib8] Johswich A. Kraft B. Wuhrer M. Berger M. Deelder A. M., 2009 Golgi targeting of Drosophila melanogaster beta4GalNAcTB requires a DHHC protein family-related protein as a pilot. J. Cell Biol. 184: 173–183 19139268 10.1083/jcb.200801071 PMC2615082

[bib9] Kleene R. Schachner M., 2004 Glycans and neural cell interactions. Nat. Rev. Neurosci. 5: 195–208 14976519 10.1038/nrn1349

[bib10] Koles K. Irvine K. D. Panin V. M., 2004 Functional characterization of Drosophila sialyltransferase. J. Biol. Chem. 279: 4346–4357 14612445 10.1074/jbc.M309912200

[bib11] Koles K. Lim J. M. Aoki K. Porterfield M. Tiemeyer M., 2007 Identification of N-glycosylated proteins from the central nervous system of Drosophila melanogaster. Glycobiology 17: 1388–1403 17893096 10.1093/glycob/cwm097

[bib12] Leonard R. Rendic D. Rabouille C. Wilson I. B. Preat T., 2006 The Drosophila fused lobes gene encodes an N-acetylglucosaminidase involved in N-glycan processing. J. Biol. Chem. 281: 4867–4875 16339150 10.1074/jbc.M511023200

[bib13] Lin D. M. Goodman C. S., 1994 Ectopic and increased expression of Fasciclin II alters motoneuron growth cone guidance. Neuron 13: 507–523 7917288 10.1016/0896-6273(94)90022-1

[bib14] Neeleman A. P. van de Eijnden D. H., 1996 Alpha-lactalbumin affects the acceptor specificity of Lymnaea stagnalis albumen gland UDP-GalNAc:GlcNAc beta-R beta 1→4-N-acetylgalactosaminyltransferase: synthesis of GalNAc beta 1→4Glc. Proc. Natl. Acad. Sci. USA 93: 10111–10116 8816760 10.1073/pnas.93.19.10111 PMC38345

[bib15] Ramakrishnan B. Qasba P. K., 2002 Structure-based design of beta 1,4-galactosyltransferase I (beta 4Gal-T1) with equally efficient N-acetylgalactosaminyltransferase activity: point mutation broadens beta 4Gal-T1 donor specificity. J. Biol. Chem. 277: 20833–20839 11916963 10.1074/jbc.M111183200

[bib16] Ramakrishnan B. Qasba P. K., 2007 Role of a single amino acid in the evolution of glycans of invertebrates and vertebrates. J. Mol. Biol. 365: 570–576 17084860 10.1016/j.jmb.2006.10.034 PMC1850938

[bib17] Ranganayakulu G. Schulz R. A. Olson E. N., 1996 Wingless signaling induces nautilus expression in the ventral mesoderm of the Drosophila embryo. Dev. Biol. 176: 143–148 8654890 10.1006/dbio.1996.9987

[bib18] Repnikova E. Koles K. Nakamura M. Pitts J. Li H., 2010 Sialyltransferase regulates nervous system function in Drosophila. J. Neurosci. 30: 6466–6476 20445073 10.1523/JNEUROSCI.5253-09.2010 PMC3354699

[bib19] Schauer R., 2009 Sialic acids as regulators of molecular and cellular interactions. Curr. Opin. Struct. Biol. 19: 507–514 19699080 10.1016/j.sbi.2009.06.003 PMC7127376

[bib20] Stolz A. Haines N. Pich A. Irvine K. D. Hokke C. H., 2008 Distinct contributions of beta 4GalNAcTA and beta 4GalNAcTB to Drosophila glycosphingolipid biosynthesis. Glycoconj. J. 25: 167–175 17876704 10.1007/s10719-007-9069-5

[bib21] Varki A., 2007 Glycan-based interactions involving vertebrate sialic-acid-recognizing proteins. Nature 446: 1023–1029 17460663 10.1038/nature05816

[bib22] Varki A., 2008 Sialic acids in human health and disease. Trends Mol. Med. 14: 351–360 18606570 10.1016/j.molmed.2008.06.002 PMC2553044

[bib23] Varki A. Schauer R., 2009 Sialic acids, pp. 199–219 Essentials of Glycobiology, edited by Varki A. R. D. Cummings J. D. Esko H. H. Freeze P. Stanley Cold Spring Harbor Laboratory Press, Cold Spring Harbor, NY

[bib24] Watanabe S. Kokuho T. Takahashi H. Takahashi M. Kubota T., 2002 Sialylation of N-glycans on the recombinant proteins expressed by a baculovirus-insect cell system under beta-N-acetylglucosaminidase inhibition. J. Biol. Chem. 277: 5090–5093 11741890 10.1074/jbc.M110548200

